# HIV-Infection, Atherosclerosis and the Inflammatory Pathway: Candidate Gene Study in a Spanish HIV-Infected Population

**DOI:** 10.1371/journal.pone.0112279

**Published:** 2014-11-10

**Authors:** Laura Ibáñez, Pablo Sebastián Velli, Roser Font, Angeles Jaén, Josep Royo, Daniel Irigoyen, Mireia Cairó, Alejandro De la Sierra, María Jesús Arranz, David Gallardo, David Dalmau

**Affiliations:** 1 Fundació Docència i Recerca MútuaTerrassa, Terrassa, Catalonia, Spain; 2 Hospital Universitari MútuaTerrassa, Terrassa, Catalonia, Spain; 3 Universitat de Barcelona, Terrassa, Catalonia, Spain; 4 Servei Veterinari de Genètica Molecular (SVGM) - Universitat Autònoma de Barcelona, Bellaterra, Catalonia, Spain; Rutgers University, United States of America

## Abstract

**Background:**

Higher prevalence of atherosclerosis and higher cardiovascular risk is observed in HIV-infected individuals. The biological mechanisms underlying these processes are unclear. Several studies have implicated genetic variants in the inflammatory genes in cardiovascular disease and in HIV natural course infection.

**Methods & Findings:**

In this study we have tested the possible association between genetic variants in several inflammatory genes and asymptomatic cardiovascular disease measured by carotid intima media thickness (cIMT) and atherosclerotic plaque presence as dependent variables in 213 HIV-infected individuals. A total of 101 genetic variants in 25 candidate genes have been genotyped. Results were analyzed using Plink and SPSS statistical packages. We have found several polymorphisms in the genes *ALOX5* (rs2115819 *p* = 0.009), *ALOX5AP* (rs9578196 *p* = 0.007; rs4769873 *p* = 0.004 and rs9315051 *p* = 0.0004), *CX3CL1* (rs4151117 *p* = 0.040 and rs614230 *p* = 0.015) and *CCL5* (rs3817655 *p* = 0.018 and rs2107538 *p* = 0.018) associated with atherosclerotic plaque. cIMT mean has been associated with *CRP* (1130864 *p* = 0.0003 and rs1800947 *p* = 0.008), *IL1RN* (rs380092 *p* = 0.002) and *ALOX5AP* (rs3885907 *p* = 0.02) genetic variants.

**Conclusions:**

In this study we have found modest associations between genetic variants in several inflammatory genes and atherosclerotic plaque or cIMT. Nevertheless, our study adds evidence to the association between inflammatory pathway genetic variants and the atherosclerotic disease in HIV-infected individuals.

## Background

Cardiovascular disease (CVD), has been identified as a major cause of death in HIV-infected people [1]–[3]. HIV-infected individuals have accelerated atherogenesis, which is associated to a high risk of suffering a cardiovascular event such as coronary artery disease (CAD), peripheral vascular disease and stroke. The prevalence of these events in HIV-infected subjects is higher than in the general population and has an earlier onset [4]–[6]. The biological mechanisms underlying such risk among HIV-infected people are unclear [7].

Several studies implicate inflammation processes in CVD [7]–[10]. Non-HIV infected cohort studies have demonstrated that markers of inflammation are strongly predictive of CVD events and mortality [8]. Inflammatory markers are elevated in HIV-infected patients in comparison with non-infected individuals [7], [11]. It has been hypothesized that this increased inflammation may be the explanation for the elevated cardiovascular risk in HIV-infected individuals [9].

Mechanisms of immune activation and inflammation have been proposed as the cause of earlier CAD in HIV [7]. HIV is thought to play a crucial role in the pathogenesis of atherosclerosis in HIV-infection [6], [11]. In addition to the high prevalence of traditional CVD risk factors in HIV-infected individuals, several factors such as immunosupression, inflammation, HIV ability to induce foam cell transformation, cumulative exposure to antiretroviral drugs, and mitochondrial and metabolic dysfunctions have been hypothesized to be involved in HIV associated atherosclerosis [11]–[13].

Studies on the inflammatory pathways have found genetic variants associated with atherosclerosis and cardiovascular risk in the general population [10]. Inflammatory marker genes such as *C-Reactive Protein (CRP)* [14], [15], *Interleucin-6 (IL6)* [10], *Interleucin-1 (IL1)* gene cluster [16], [17], *Interleucin-18 (IL18)* [10], *Interleucin-8 (IL8)* [10], *Tumor Necrosis Factor (TNF)* [10], *Lymphotoxin-α (LTA)* [10], *Fractalkine Receptor (CX3CR1)* [18], [19], *Chemokine Receptor 5 (CCR5)* [20], *Chemokine Receptor 2 (CCR2)* and the 5-lipoxygenase (5-LO) pathway genes [10], [21] have been associated with cardiovascular events. Regarding HIV-infected patients, the most important investigation is a Genome Wide Association Study (GWAs) conducted by Shrestha et al. [22] that related two variants in the gene *Ryanodine Receptor 3* (*RYR3)* with greater carotid Intima Media Thickness (cIMT), a surrogate marker of atherosclerosis. This finding has been replicated by the same authors in a later study [23], but there is no independent study confirming this result.

The immunological and inflammatory pathways have many shared genes that may interact in the pathogenesis of atherosclerosis in HIV-infected individuals. The aim of this study was to assess the implication of genetic variants in relevant inflammatory genes in the atherosclerotic disease of HIV infected subjects.

## Materials and Methods

### Study population

We performed a cross-sectional study with 213 Spanish Caucasians HIV-infected individuals attended in Hospital Universitari MútuaTerrassa (Terrassa, Catalonia, Spain). This project was approved by the local ethics committee (Comité ético de Investigación Clínica del Hospital Universitario MútuaTerrassa - Approval number: EO/0915). All Participants gave written informed consent for genetic testing. At the time of enrolment, demographic, clinical and biochemical variables were collected from each patient by interviews and from medical notes. Simultaneously ultrasonographic measures (cIMT and atherosclerotic plaque presence) were performed.

### Carotid artery Ultrasound

Carotid Intima Media Thickness (cIMT) is the most widely used surrogate marker of atherosclerosis. It relies on the fact that the presence of atherosclerosis in one vascular bed will correlate with the atherosclerosis in another vascular bed. High-resolution B-mode ultrasound of the carotid arteries has been well validated in the non infected population as a surrogate marker of cardiovascular risk [24].

cIMT measurements and atherosclerotic plaque presence were obtained for each patient using a B-mode ultrasound recording with a 7 to 14-MZ transductor. For the imaging studies, patients were placed in the supine position with their head in the midline position and tilted slightly upwards, and the heart in systole. For each participant, a total of eight measures were obtained: left and right carotid primitive and bulb region in both proximal and distal walls. The cIMT value was defined as the mean of all values excluding those corresponding to atherosclerotic plaque thickness. According to Manheim cIMT consensus, atherosclerotic plaque presence is defined as a focal structure encroaching into the arterial lumen by at least 0.5 mm or 50% of the surrounding IMT value [25]. It was recorded as a bimodal variable (yes/no). All measures were performed by the same operator with an experimental intra-variability of 4.7%.

### Genotyping

Genomic DNA was extracted from whole blood using the QIAamp DNA Blood Mini kit (Qiagen, Izasa, Barcelona, Spain) following the manufacturer's instructions. Genotyping was performed using Competitive Allele PCR methodology (KBioscience, Hoddesdon, United Kingdom). Genes from the inflammatory pathway were selected. A total of 101 genetic variants or single nucleotide polymorphisms (SNPs) in 25 candidate genes were tested (Table 1). TagSNPs were selected from HapMap for maximum coverage of the genes (Selection criteria: R^2^>0.8 and minor allele frequency (*maf*)>0.05). We also included some relevant SNPs from other studies in HIV and non-HIV infected populations.

**Table 1:** Genotyped SNPs.

Chromosome	Gene	Genotyped SNPs
1	*CRP*	rs1205; rs1130864; rs1800947
2	*IL1B*	rs55778004; rs1143634; rs1143633; rs1143627*; rs16944*
	*IL1RN*	rs3087263; rs380092; rs431726; rs452204; rs4252019; rs315952; rs4252041
3	*CCR2*	rs3762823; rs1799864
	*CCR5*	rs333*
	*IL12A*	rs2243123; rs583911
4	*IL8*	rs4073*; rs2227306
	*CXCL1*	rs4074
	*CXCL2*	rs9131; rs3806792*
	*CXCL10*	rs8878; rs11548618*
	*IL2*	rs2069778; 2069777
6	*LTA*	rs1800683; rs2239704; rs909253; rs2229094; rs2229092
	*TNF*	rs4248160*; rs3093662
7	*IL6*	rs1800795; rs2069833; 2069840; rs1554606
9	*IL33*	rs4742170; rs7019575; rs7037276; rs1412420; rs7047921; rs1332290
10	*CXCL12*	rs1801157; rs2236533; rs2236534; rs2839693; rs10793538; rs3780891; rs7092453
	*ALOX5*	rs12783095; rs3824612; rs934187; rs7918542; rs7099684; rs2115819; rs10900213; rs11239523; rs12264801; rs1565096; rs1487562; rs3780914
11	*IL18*	rs3882891; rs5744258; rs5744247; rs795467; rs2043055
13	*ALOX5AP*	rs9579645; rs9579646; rs4075131; rs9578196; rs4293222; rs12429692; rs4769873; rs9315045; rs4503649; rs3885907; rs10162089; rs4254165; rs17245204; rs9579648; rs10507393; rs9315048; rs9315051; rs3935644; rs4769060
15	*RYR3*	rs2229116*
16	*CX3CL1*	rs170364; rs170361; rs4151117; rs614230
17	*CCL2*	rs1024611; rs3760396
	*CCL5*	rs3817655; rs2280789; rs2107538
	*CCL3*	rs1719134
	*CCL4*	rs1719147

*Selected from previous published studies.

### Statistical analysis and quality control

Statistical analyses were performed using the G*Power Calculator [26], SPSS 20 (SPSS, Chicago, IL) and Plink 1.07 [27] statistical packages. The study had an 80% power (considering α = 0.05 and β = 0.95, two-sided) to capture the effect of SNPs with *maf*≥0.1 and Odds ratio (OR)≥2. For rarer alleles (*maf<0.1*), the study had a 75% power to capture genetic effects with OR≥3. For continuous variables the study had a 95% power (considering α = 0.05 and β = 0.95, two-sided) to capture the effect of SNPs with *maf*≥0.1.

Genotype call rate, Hardy-Weinberg equilibrium (HWE) and *maf* was assessed for each SNP using Plink 1.07. Those SNPs with call rates<95% or *maf*<0.01 and individuals with more than 5% missing genotypes were excluded from the analyses.

Chi square analyses or fisher exact tests (for those alleles with less than 5 counts in any group) were used to investigate the possible association of atherosclerotic plaque with genetic variants. cIMT variable was recorded as a continuous variable and it was normally distributed. Linear regression analyses were used to compare cIMT values with gene alleles.

Age, abdominal obesity, metabolic syndrome, hypertension, diabetes, total cholesterol, non-HDL cholesterol, LDL cholesterol and triglycerides were significantly associated with atherosclerotic plaque presence (p<0.05 in all cases). Age, hypertension, diabetes, dyslipidemia, total cholesterol, non-HDL cholesterol and LDL cholesterol were significantly associated with cIMT (p<0.05 in all cases).

Lipid values were highly correlated (correlation coefficient>0.75 in all cases). Only dyslipidemia was included as a clinical adjusting variable in the regression models. Metabolic syndrome was collinear with the variables diabetes, dyslipidemia, abdominal obesity and hypertension. Metabolic syndrome was not included as a clinical variable in the regression model for atherosclerotic plaque presence.

For atherosclerotic plaque presence, age, abdominal obesity, hypertension, diabetes and dyslipidemia were included in logistic regression analyses as adjusting clinical variables for all the findings. For cIMT, age, hypertension, diabetes, and dyslipidemia were included in the regression analyses as adjusting clinical variables for all the findings. For the importance of HAART in the vascular pathogenesis, it was included in both adjustments.

## Results

### Study Population

Demographic, lipid profile and HIV baseline characteristics of the study population are shown in Table 2. Mean age was 45.34 (SD±8.20) years. Males constituted 77.9% of the samples. 82.2% of the individuals were receiving Highly Active Antiretoviral Therapy (HAART) at the time of the study. Atherosclerotic plaque presence (indicator of atherosclerotic lesion [28]) and cIMT means (indicator of arterial remodelling [29]) were the dependent variables investigated as markers of CVD. The study population was classified in two groups according to the bimodal variable atherosclerotic plaque presence. Baseline characteristics are shown in Table 1. Patients with atherosclerotic plaque presence conformed 39% of the cohort (n = 83), with 73.5% males and a mean age of 49.67 (SD±8.38) years. Patients without atherosclerotic plaque had a mean age of 42.58 (SD±6.79) years and 80.8% were males.

**Table 2:** Baseline characteristics of sample population.

Variable	Value	Atherosclerotic Plaque Presence	
		Yes (n = 83)	No (n = 130)
Age, years±sd	45.34±8.20	49.67±8.38	42.58±6.79
Males, n (%)	166 (77.9)	61 (73.5)	105 (80.8)
Body Mass Index*, Kg/m^2^±sd	24.08±3.76	24.11±4.09	24.06±3.56
Abdominal Obesity†, n (%)	26 (12.2)	15 (18.3)	11 (8.6)
Metabolic Syndrome‡, n (%)	16 (7.5)	10 (12.5)	6 (4.7)
Hypertension§, n (%)	20 (9.4)	14 (17.7)	6 (4.7)
Diabetes Mellitus||, n (%)	8 (3.8)	7 (8.4)	1 (0.8)
Dyslipidemia#, n (%)	59 (27.7)	28 (35.0)	31 (24.0)
Atherosclerosis Characteristics			
cIMT mean, mm±sd	0.89±0.21	1.01±0.21	0.81±0.17
Plaque presence, n (%)	83 (39.0)	83 (100)	0 (0)
Smoking Habits			
Smokers, n (%)	150 (70.4)	61 (74.4)	89 (70.1)
Non Smokers, n (%)	59 (27.7)	21 (25.6)	38 (29.9)
Lipid profile			
Total Cholesterol, mg/dl±sd	181.41±43.13	193.01±47.70	174.01±38.33
LDL Cholesterol, mg/dl±sd	111.70±49.15	118.81±49.23	107.15±48.75
HDL Cholesterol, mg/dl±sd	46.97±16.17	47.98±16.24	46.32±16.16
Non-HDL Cholesterol, mg/dl±sd	134.67±42.28	145.03±48.16	128.05±36.75
Triglycerides, mg/dl±sd	148.09±92.78	160.76±84.70	140.00±97.04
HIV Characteristics			
Time Infected, years±sd	11.97±7.62	13.26±7.50	11.10±7.62
Hepatitis C Coinfection, n (%)	102 (47.9)	45 (54.9)	57 (44.5)
Antiretroviral Therapy**, n (%)	175 (82.2)	72 (87.8)	103 (80.5)
PIs, n(%)	62 (29.1)	24 (30.0)	38 (30.4)
nRTIs, n(%)	166 (77.9)	69 (86.2)	97 (77.0)
NNRTIs, n(%)	101 (47.7)	42 (52.5)	59 (46.8)
Entry Inhibitors, n(%)	2 (0.9)	1 (1.2)	1 (0.8)
Integrase Inhibitors, n(%)	12 (5.6)	7 (8.8)	5 (4.0)
Previous Antiretroviral Therapies, n (%)	167 (78.4)	68 (82.9)	99 (77.3)
CD4+ cell count, cells/µl±sd	581.25±339.53	610.10±359.48	562.83±326.23
CD4+ nadir cell count, cells/µl±sd	301.74±222.79	310.09±242.74	296.39±209.88
Viral Load> 19 copies/ml, n (%)	71 (33.3)	23 (27.7)	48 (36.9)
Viral Load, copies/ml (95%IQR)	95759.65±203731.45	14352.03±61553.87	43494.40±152702.15
CDC Stage			
A, n (%)	143 (67.1)	59 (72.8)	84 (67.2)
B, n (%)	13 (6.1)	3 (3.7)	10 (8.0)
C, n (%)	50 (23.5)	19 (23.5)	31 (24.8)
Diagnosed AIDS, n (%)	92 (43.2)	37 (45.1)	55 (44.0)
Risk Group			
Drug Users, n (%)	78 (36.6)	35 (50.7)	43 (40.6)
Sexual Transmission, n (%)	91 (42.7)	32 (46.4)	59 (55.7)
Others, n (%)	6 (2.8)	2 (2.9)	4 (3.8)

*Body Mass Index (BMI): the individual's body mass divided by the square of their height.

† Abdominal Obesity: waist circumference>102 cm in men and>88 cm in women.

‡ Metabolic Syndrome was defined as having two or more of the following characteristics: Diabetes Mellitus^||^; Blood Pressure ≥140/90 mmHg; Dyslipidemia^#^; Abdominal Obesity^†^ and hypertension^§^.

§ Hypertension was: systolic blood pressure ≥140 and/or diastolic blood pressure ≥90 mmHg and/or antihypertensive treatment.

|| Diabetes Mellitus: fasting glucose levels ≥126 mg/dl, and/or having diabetes symptoms and glucose levels ≥200 mg/dl in a random determination and/or diabetes treatment.

# Dyslipidemia: Total Cholesterol ≥240 mg/dl, and/or HDL Cholesterol ≤35 mg/dl, and/or Triglycerides ≥200 mg/dl and/or lipid lowering drugs

**Antiretroviral Therapy: PIs (Protease Inhibitors); nRTIs (Nucleoside Reverse Transcriptase Inhibitors); NNRTIs (Non-Nucleoside Reverse Transcriptase Inhibitors); Entry Inhibitors and Integrase Inhibitors.

### Quality Control

#### Hardy-Weinberg Equilibrium

All SNPs were in HWE except for *IL1B* rs16944 (*p* = 0.044), *IL1RN* rs380092 (*p* = 0.006), *CXCL12* rs2236533 and rs2236533 (*p = *0.040 and *p = *0.029 respectively) and *ALOX5* rs3824612 (*p* = 0.025).

#### SNP Quality control

Three of the genotyped SNPs were excluded from the analysis, because of a call rate<95% (*IL1RN* rs3087263, *CX3CR1* rs2669845 and *IL6* rs2069833) and two because of a *maf*<0.01 (*CXCL10* rs11548618 and *TNF* rs4248160). Two SNPs (*IL1B* rs55778004 and *IL1RN* rs4252019) were monomorphic in our population. The remaining 94 SNPs were included in the analysis.

### Single Marker Analysis

#### Atherosclerotic Plaque Presence Analysis

Single marker analyses by Chi-square revealed 12 associations with atherosclerotic plaque presence (Table 3). *IL1RN* rs4252041 rare allele was associated with atherosclerotic plaque presence (*p* = 0.027, OR = 2.40). *CXCL2* rs9131 and rs3806792 rare alleles were associated with atherosclerotic plaque absence (*p* = 0.035, OR = 0.65 and *p* = 0.026, OR = 0.63 respectively) whereas *ALOX5* rs2115819 was associated with atherosclerotic plaque presence (*p* = 0.008, OR = 1.73). Three *ALOX5AP* SNPs were associated with atherosclerotic plaque absence: rs9578196 (*p* = 0.014, OR = 0.45); rs4769873 (*p* = 0.011, OR = 0.40) and rs9315051 (*p* = 0.001, OR = 0.27).

**Table 3:** Summary of single marker analyses in relation to atherosclerotic plaque presence.

Chr	Gene	SNP	ALLELE COUNT	Unadjusted			Adjusted**		
			Minor allele (MAF Aff/MAF Unaff*)	p value	OR	CI 95%	p value	β	CI 95%
2	*IL1RN*	rs4252041	T (0.10/0.04)	0.027	2.40	1.08–5.30	*ns*	*-*	*-*
4	*CXCL2*	rs9131	G (0.37/0.47)	0.035	0.65	0.43–0.97	*ns*	*-*	*-*
4	*CXCL2*	rs3806792	T (0.36/0.47)	0.026	0.63	0.42–0.95	*ns*	*-*	*-*
10	*ALOX5*	rs2115819	C (0.50/0.37)	0.008	1.73	1.15–2.58	0.009	2.03	1.19–3.47
13	*ALOX5AP*	rs9578196	T (0.08/0.16)	0.014	0.45	0.23–0.86	0.007	0.33	0.14–0.73
13	*ALOX5AP*	rs4769873	T (0.06/0.14)	0.011	0.40	0.19–0.83	0.004	0.25	0.10–0.65
13	*ALOX5AP*	rs9315051	G (0.04/0.14)	0.001	0.27	0.12–0.63	0.0004	0.15	0.05–0.43
16	*CX3CL1*	rs170361	A (0.13/0.20)	0.040	0.57	0.33–0.98	*ns*	*-*	*-*
16	*CX3CL1*	rs4151117	G (0.15/0.24)	0.024	0.55	0.33–0.93	0.040	0.52	0.28–0.97
16	*CX3CL1*	rs614230	C (0.27/0.39)	0.013	0.59	0.38–0.89	0.015	0.54	0.33–0.88
17	*CCL5*	rs3817655	A (0.23/0.15)	0.035	1.72	1.04–2.85	0.018	1.96	1.12–3.42
17	*CCL5*	rs2107538	T (0.23/0.15)	0.033	1.71	1.04–2.80	0.018	1.93	1.12–3.31

*maf: minor allele frequency/Aff: Affected/Unaff: Unaffected

**Adjusted by clinical variables: Age, Abdominal Obesity, Metabolic Syndrome, Hypertension, Diabetes, Dyslipidemia and Antiretroviral Therapy

*CX3CL1* rs170361, rs4151117 and rs614230 were associated with atherosclerotic plaque absence (*p* = 0.040, OR = 0.57; *p* = 0.024, OR = 0.55 and *p* = 0.013, OR = 0.59 respectively). Finally, two *CCL5* SNPs were associated with atherosclerotic plaque presence: rs3817655 (*p* = 0.035, OR = 1.72) and rs2107538 (*p* = 0.033, OR = 1.71).

Single marker analyses by logistic regression using age, abdominal obesity, hypertension, diabetes, dyslipidemia and HAART as adjusting clinical variables showed 8 positive associations.

*ALOX5* rs2115819 rare allele C was associated with atherosclerotic plaque presence (*p* = 0.009, β = 2.03). Three *ALOX5AP* SNPs were associated with atherosclerotic plaque absence: rs9578196 (*p* = 0.007, β = 0.33); rs4769873 (*p* = 0.004, β = 0.25); and rs9315051 (*p* = 0.0004, β = 0.15).

*CX3CL1* rs4151117-G and rs614230-C were associated with atherosclerotic plaque absence (*p* = 0.040, β = 0.52 and *p* = 0.015, β = 0.54 respectively). Two *CCL5* SNPs were associated with atherosclerotic plaque presence: rs3817655 (*p* = 0.018, β = 1.96) and rs2107538 (*p* = 0.018, β = 1.93).

#### Carotid Intima Media Thickness Analysis

Single marker analysis by linear regression showed 6 associations with cIMT. (Table 4). *CRP* rs1130864 was associated with smaller cIMT values (*p* = 0.006, β = −0.06) whereas rs1800947 was associated with greater cIMT (*p* = 0.028, β = 0.09). *IL1RN* rs380092 was not in HWE, but we found it associated with smaller cIMT (*p* = 0.019, β = −0.06). *IL8* rs2043055 and *ALOX5AP* rs3885907 showed association with greater cIMT (*p* = 0.040, β = 0.04 and *p* = 0.030, β = 0.05 respectively). Finally, *CX3CL1* rs170361 showed a trend towards association with smaller cIMT (*p* = 0.049, β = −0.05).

**Table 4:** Summary of single marker analyses in relation to cIMT.

Chr	Gene	SNP	ALLLELE COUNT	Unadjusted			Adjusted**		
			MAF	p value	β	CI 95%	p value	β	CI 95%
1	*CRP*	rs1130864	Minor Allele T–0.34	0.006	−0.06	−0.10–−0.02	0.0003	−0.07	−0.11–−0.03
1	*CRP*	rs1800947	Minor Allele C–0.06	0.028	0.09	0.01–0.17	0.008	0.10	0.03–0.17
2	*IL1RN*	rs380092*	Minor Allele T–0.25	0.019	−0.06	−0.11–−0.01	0.002	−0.07	−0.12–−0.03
11	*IL18*	rs2043055	Minor Allele G–0.42	0.040	0.04	0.002–0.08	*ns*	*-*	*-*
13	*ALOX5AP*	rs3885907	Minor Allele C–0.41	0.030	0.05	0.005–0.09	0.02	0.05	0.01–0.08
16	*CX3CL1*	rs170361	Minor Allele A–0.17	0.049	−0.05	−0.10–−0.001	*-*	*-*	*-*

*Not in HWE.

**Adjusted by clinical variables: Age, Hypertension, Diabetes, Dyslipidemia and Antiretroviral Therapy.

Single marker analyses by linear regression using age, hypertension, diabetes mellitus, dyslipidemia and HAART as adjusting clinical variables showed 4 significant associations. Two *CRP* SNPs showed association with cIMT: rs1130864 with smaller cIMT (*p* = 0.0003, β = -0.07) and rs1800947 with greater cIMT (*p* = 0.008, β = 0.10). *IL1RN* rs380092 rare allele T was associated with smaller cIMT (*p* = 0.002, β = −0.07). Finally *ALOX5AP* rs3885907 allele C was associated with greater cIMT (*p* = 0.02, β = 0.05).

## Discussion

In this study we have tested the possible association between genetic variants in several inflammatory genes and CVD measured by cIMT and atherosclerotic plaque presence in 213 HIV-infected individuals. We have found several polymorphisms in the *ALOX5, ALOX5AP, CX3CL1* and *CCL5* genes significantly associated with atherosclerotic plaque, whereas cIMT mean has been associated with *CRP, IL1RN* and *ALOX5AP* genetic variants.

### Atherosclerotic Plaque

The *ALOX5* rs2115819-C allele was associated with atherosclerotic plaque presence and the *ALOX5AP* rs9578196-T, rs4769873-T and rs9315051-G alleles with atherosclerotic plaque absence. Genetic variants in *ALOX5* and *ALOX5AP* have been previously linked to CVD in non-HIV infected populations [21]. These two genes encode for two important proteins of the 5-LO pathway which has been previously linked to atherosclerosis [21]. Knock-out mice of *ALOX5* showed less atherosclerotic plaque formation [21]. Studies in HIV-infected cultured cells have shown a diminished function of 5-LO pathway due to gp120 HIV-protein presence [30], [31]. Our results, showing that ALOX5 and ALOX5AP may influence CVD in HIV patients, contribute to the growing evidence on the relevance of these genes on CVD.

The *CX3CL1* rs614230-C allele was found associated with absence of atherosclerotic plaque. This allele has been previously associated with smaller cIMT in a German non-HIV infected subjects (CAPS cohort) although this finding was not replicated in a French cohort (3C cohort) [18]. To our knowledge, the association found between *CX3CL1* rs4151117-G allele and atherosclerotic plaque absence is a novel finding. CX3CL1 has been implicated in atherosclerotic plaque formation initial process [18], [19]. *CX3CR1* (CX3CL1 receptor) knockout mice and animal treated with CX3CR1 inhibitors show reduced atherosclerotic plaque formation [32], [33]. The functional effects of the CX3CL1 rs614230 or rs4151117 are not known. However, these SNPs are located near a frame shift mutation that may diminish protein functionality. Our results seem to agree with the reported atheroprotective effects of CX3CL1 reduced activity.

The *CCL5* rs3817655-A and rs2107538-T alleles were found associated with atherosclerotic plaque presence. The *CCL5* rs2107538 polymorphism has been previously associated with higher plasma concentrations of CCL5 and increased risk of MI in Korean CAD patients [34] and Han Chinese MI patients [15]. Our results seem to agree with these findings as the T allele associated with plaque presence in our study is also associated with MI risk. The *CCL5* rs3817655 finding is a novel association that has not been previously described. This SNP is near to two mutations that encode for new stop codons and truncated proteins. Our findings may reflect the functional effect of these mutations but further studies are required to confirm it.

### Carotid Intima Media Thickness

The *CRP* rs1130864-T allele was found marginally associated with smaller cIMT and the rs1800947-C allele with greater cIMT. These results are in the opposite direction that in previous studies. The *CRP* rs1130864-T allele was found associated with increased CRP plasma levels [14], which are considered a marker of cardiovascular risk [35], [36], and have been linked to a faster cIMT progression in HIV-infected individuals [37]. The *CRP* rs1800947-C allele have been previously linked to decreased CRP plasma levels [15], [38], which are known to be atheroprotective. Although the functional consequences of these SNPs are not known, both of them are in regulatory regions. The presence of HIV could interact with the functionality of these genetic variants explaining the discrepancy between our findings and those published previously. These findings need to be replicated in independent cohorts to confirm the direction of the associations.

The *IL1RN* rs380092-T allele was found associated with smaller cIMT, although in previous studies has been associated with increased risk of MI [16], [17], [39]. The *IL1RN* rs380092 variant is located approximately 200 bp up-stream of another variant, rs121913161, which encodes for a new stop codon that significantly reduces IL1RN plasma levels. IL1RN has been suggested to be a proatherogenic cytokine [40] and diminished levels of IL1RN might be atheroprotective [41]. This would agree with our findings. However, this result needs to be repeated as this SNP was not found in HWE.

In studies performed by Shrestha et al. on American HIV-infected patients, an association was found between the *RYR3* rs2229116 polymorphism and cIMT [22]. However, we were not able to replicate this finding. Aside from a false negative result, the discrepancy could be explained by the methodological differences in cIMT measures and/or different allele frequencies between American and Spanish populations.

This study has several limitations. Our cohort has a limited sample size, although it is very well characterized. High prevalence of cardiovascular risk traditional factors and the bias observed in HIV-infected populations should also be considered as a limitation and a possible source of error in our findings. The lack of an HIV-negative control group prevented drawing any conclusions about the contribution of HIV infection to early atherosclerosis. However, studies have suggested that the biological processes involved in HIV-related atherosclerosis are different from the ones observed in non-infected individuals. Moreover, it seems that the atherosclerotic plaque structure and characteristics are also different [42]. Finally, carotid ultrasonography measures are conditioned by its inter and intra-variability.

In summary, in this study we have found modest associations between genetic variants in several inflammatory genes and cardiovascular risk markers in an HIV-infected population. They need to be confirmed in a larger population and functional studies are needed to elucidate the consequences of these genetic changes. Nevertheless, our study adds evidence to the association between inflammatory pathway genetic variants and the atherosclerotic disease in HIV-infected individuals.
